# Comparison Between Automated Office Blood Pressure Measurements and Manual Office Blood Pressure Measurements—Implications in Individual Patients: a Systematic Review and Meta-analysis

**DOI:** 10.1007/s11906-020-01118-1

**Published:** 2021-01-15

**Authors:** Yacong Bo, Kin-On Kwok, Kareen Ka-Yin Chu, Eppie Yu-Han Leung, Chun Pong Yu, Samuel Yeung-Shan Wong, Eric Kam-Pui Lee

**Affiliations:** 1 Jockey Club School of Public Health and Primary Care, Faculty of Medicine, The Chinese University of Hong Kong, Hong Kong SAR, China; 2 Department of Continuing Education, University of Oxford, Oxford, UK; 3 Li Ping Medical Library, The Chinese University of Hong Kong, Hong Kong SAR, China; 4 Room 402, School of Public Health, Prince of Wales Hospital, Shatin, Hong Kong

**Keywords:** Automated office blood pressure, Ambulatory blood pressure measurement, Manual office blood pressure, Meta-analysis, White-coat hypertension, Masked hypertension

## Abstract

**Purpose of Review:**

Automated office blood pressure (AOBP) measurements may provide more accurate estimation of blood pressure (BP) than manual office blood pressure (MOBP) measurements. This systematic review investigated the diagnostic performance of AOBP and MOBP using ambulatory blood pressure measurement (ABPM) as reference. Several databases including MEDLINE, Embase, Scopus, and China Academic Journals were searched. Data were extracted, double-checked by two investigators, and were analysed using a random effects model.

**Recent Findings:**

A total of 26 observational studies were included. The mean systolic/diastolic BP obtained by AOBP was not significantly different from that obtained by ABPM. The sensitivity and specificity of AOBP to detect elevated BP were approximately 70%. Fewer participants had white-coat hypertension on AOBP measurement than on MOBP measurement (7% versus 14%); however, about 13% had masked hypertension on AOBP measurement. The width of the limit of agreement comparing (i) AOBP and ABPM and (ii) MOBP and ABPM was comparable.

**Summary:**

AOBP may reduce the rate of the observed white-coat effect but undermine masked hypertension. The current recommendation, however, is limited by the absence of high-quality studies and the high heterogeneity of our results. More high-quality studies using different AOBP machines and in different population are therefore needed.

**Supplementary Information:**

The online version contains supplementary material available at 10.1007/s11906-020-01118-1.

## Introduction

Hypertension (HT) is the most common chronic disease worldwide, affecting 30% of the adult population [1, 2]. Due to its high prevalence and related complications, HT poses a substantial economic burden on healthcare systems [3].

The diagnosis and treatment of HT depend exclusively on accurate BP assessment [4]. Out-of-office measurements such as ambulatory BP monitoring (ABPM) and home BP monitoring (HBPM) are superior to office BP measurements in predicting cardiovascular complications and mortality. Nevertheless, the role of office BP should not be undermined [5, 6]. Repeating ABPM is expensive and not feasible, and the reporting bias and poor patient measurement techniques can be problematic when HBPM is employed. Furthermore, almost all landmark randomised controlled trials (RCTs) investigating HT treatments utilise office BP as the primary endpoint [7, 8]. RCTs have also shown that an improvement in office BP values can reduce cardiovascular events and/or deaths [8].

Clinically, manual office BP (MOBP) measurements involve BP measurements performed by doctors or clinical staff using a mercury, aneroid, or electronic oscillometric sphygmomanometer and are prone to both white-coat and masked effects [9]. Moreover, in daily practice, healthcare workers have poor adherence to the recommended BP measurement techniques (e.g. talking during BP measurements) and, thus, introduce further measurement bias [10]. To improve the accuracy of BP measurements in office settings, a BP measurement method called automated office blood pressure (AOBP) measurement was developed and is recommended in Canada [11]. AOBPs are electronic oscillometric sphygmomanometers (including BpTRU [VSM MedTech, Coquitlam, Canada], Omron HEM-907 [OMRON, Tokyo, Japan], and Microlife WatchBP Office [Microlife, Heerbrugg, Switzerland]) that automatically and repeatedly measure BP 3–5 times in clinical settings—typically at 1-min intervals (depending on the machine models)—in a quiet room and provide the mean of consecutive BP measurements [11]. As BP readings can be taken without healthcare professionals, AOBP should theoretically decrease the rate of observed white-coat effect and improve the accuracy of BP measurement by avoiding talking during BP measurements. Despite multiple measurements obtained by AOBP machines, the time required could be similar to that with MOBP because patients may not necessarily rest for 5 min prior to AOBP measurement [12, 13]. Recent meta-analyses determined that mean BPs obtained by AOBP measurement were similar to those obtained by daytime ABPM (regarded as the reference standard of clinical BP measurements) and HBPM; additionally, mean BPs from MOBP were substantially higher than those obtained by AOBP, HBPM, and ABPM [14–16].

Although mean BP readings obtained by AOBP measurement and ABPM (when analysed as a group of patients) were similar, it is unclear if the diagnostic performance of AOBP measurement is superior to that of MOBP measurement as they have different cut-off values for elevated BP (MOBP, ≥ 140/90 mmHg; AOBP, ≥ 135/85 mmHg) [12, 17]. Furthermore, white-coat and masked HTs are still observed when BP is measured using AOBP and BP values from AOBP and ABPM can be significantly different in individual patients [18, 19]. Until now, no systematic review or meta-analysis has compared the diagnostic accuracy, including sensitivity and specificity, of AOBP and MOBP.

Although this review originally aimed to assess the mean difference in BP values obtained by AOBP and MOBP measurements (when daytime ABPMs are used as reference), these analyses were published soon after the review was registered [15, 16]. Using the pre-specified scope of searching and besides conducting the per-specified analyses, this study further explored and compared diagnostic performance of AOBP and MOBP, using ABPM as the reference standard. This included meta-analysing data and describing (i) sensitivity and specificity of AOBP and MOBP to detect elevated BP, (ii) limit of agreement (LOA) between AOBP/MOBP and ABPM, and (iii) the proportion of participants who were wrongly categorised depending on AOBP/MOBP (including white-coat and masked HT). Accordingly, we hypothesised that AOBP would (i) have a higher sensitivity and specificity for diagnosing elevated BP, (ii) have a narrower LOA, and (iii) have less white-coat or masked effect than MOBP.

## Methods

### Study Eligibility

All prospective and retrospective observational studies in which the same group of participants, who were at least 18 years of age (with or without HT), undergoing all AOBP, traditional BP measurement and ABPM, were included. Inclusion criteria for studies were reported raw BP or mean BP readings and only those published in English and Chinese. Studies that included pregnant patients and those with autonomic neuropathy or atrial fibrillation were excluded because the hemodynamic status of these patients would be unstable and any differences in BP between various BP measurement methods might be due to underlying conditions. Animal studies, commentaries, and reviews were also excluded. This study was registered in PROSPERO (CRD42019118790) on 4 January 2019 (available from http://www.crd.york.ac.uk/PROSPERO/display_record.php?ID=CRD42019118790).

Because abstracts presented at major HT conferences were published in international peer-reviewed journals, the search strategies employed were able to pick up any study that had not been published (e.g. Journal of Hypertension for Annual European Society Hypertension Conference). When only abstracts were available, the authors were contacted as possible for any published report/article.

### Search Strategy

As previous meta-analyses only included studies published in the English language, we deliberately included Chinese databases in the literature search. The databases Ovid Medline, Embase, Scopus, and China Academic Journals Full-text Database were searched for articles published since 2001, when the first AOBP machine was validated until 28 February 2020. Keywords such as ‘ambulatory blood pressure’, ‘automated office blood pressure’, ‘automated oscillometric blood pressure’, ‘BpTRU’, ‘WatchBP Office’, and/or ‘HEM-907’ were used as the search terms. The search was limited to studies involving adults only, and the publication language was restricted to English and Chinese. The detailed search strategies used for these different databases are shown in Online Resource 1.

### Study Screening and Data Extraction

Studies from the search were all inputted into the Covidence program (Covidence systematic review software, Veritas Health Innovation, Melbourne, Australia; available at www.covidence.org). Two out of the three investigators (EKP, KC, EL) independently assessed the eligibility of studies by screening the titles/abstracts and subsequently the full texts in the Covidence program. Any differences were successfully resolved by consensus. Authors were enquired and e-mailed about the possibility of duplicated data when this was suspected.

Data were extracted by EKP and double-checked by at least one more investigator (BY or KC). Discrepancies were compared and resolved. Despite the best efforts to contact authors in order to obtain full texts when only the abstract was available, we received no responses. Thus, available data were extracted from abstracts directly whenever possible.

The following data were extracted from the included studies: sample size; year of publication of the study; country where the study was conducted; participants’ demographic details; method, details, and BP values with respect to AOBP/ABPM/MOBP measurements; lower and upper LOA using Bland-Altman method; sensitivity/specificity of AOBP and MOBP to detect elevated BP; and proportion of patients diagnosed with masked or white-coat HT on AOBP or MOBP measurement.

### Quality Assessment

The quality assessment was based on QUADAS-2, which was developed for systematic reviews of diagnostic accuracy studies and could be modified to fit in individual systematic reviews, as recommended by the authors [20]. This was expanded to enhance the assessment of quality for our reference (daytime ABPM) and index tests (office BP and AOBP). The detailed criteria are presented in Online Resource 2. The study was at low risk of bias only when the following questions were not concerned, while all other studies were categorised under ‘unknown risk/high risk of bias’. The quality assessments were conducted by EKP and were double-checked by EL.

### Analysis

All meta-analysis was conducted using Stata (StataCorp. 2017. *Stata Statistical Software: Release 15*. College Station, TX: StataCorp LLC), unless otherwise stated.

The outcome measures that were originally planned and registered in PROSPERO were the weighted mean difference in SBP and DBP between AOBP measurement and ABPM and between MOBP measurement and ABPM. The weighted mean differences in individual studies were pooled using a random effects model. The random effects model was used because significant clinical heterogeneity was predicted due to difference in ABPM machines, measurement algorithms, and populations in different studies. Heterogeneity across studies was assessed using *I*^2^ statistics, whereas publication bias was evaluated by visual examination of the funnel plot and Egger’s test. *p* values were 2-tailed and a *p* value < 0.05 was considered statistically significant. Subgroup analysis was pre-specified for AOBP (i) study quality, (ii) ethnicity, and (iii) different AOBP models. For MOBP, subgroup analysis included (i) persons who were responsible for BP measurement, (ii) ethnicity, and (iii) manual measurements versus semi-automatic BP machines. An SBP of at least 130 mmHg on AOBP measurement and whether the AOBP was unattended were found to be important as per the latest systematic reviews [15, 21], which were also conducted post hoc. Because one of the included studies enrolled patients receiving peritoneal dialysis (*n* = 17) [22], a sensitivity analysis that excluded this study was conducted, whenever appropriate.

Other analyses included meta-analysis of sensitivity and specificity, for which the *metandi* command in Stata was used, as well as proportions (including proportions of participants with white-coat HT and participants with masked HT), which were analysed using the *metaprop* command in Stata. Other secondary analyses included meta-analysis of Bland-Altman statistics, the method of which has been published and was analysed by R (R Foundation for Statistical Computing, Vienna, Austria) [23].

A BP of 135/85 mmHg was used as the cut-off for ABPM and AOBP, whereas a BP of 140/90 mmHg was used as the cut-off for MOBP because these were the most widely adopted values [24]. White-coat HT was defined as normal BP detected on ABPM but elevated BP on AOBP/MOBP measurement. Masked HT was defined as elevated BP on ABPM but normal BP on AOBP/MOBP measurement. If the same study had involved different persons to obtain the MOBP, readings obtained by doctors were used.

## Results

### Search Results and Characteristics of the Included Studies

**Fig. 1:**
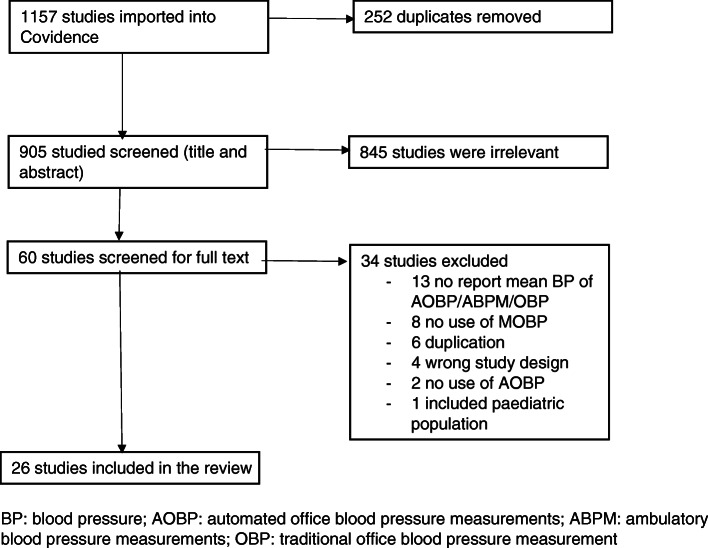
PRISMA chart. BP: blood pressure; AOBP: automated office blood pressure measurements; ABPM: ambulatory blood pressure measurements; OBP: traditional office blood pressure measurement

The PRISMA chart is shown in Fig. 1. Altogether, 26 studies involving 5407 participants were included. Details of the included studies can be found in Online Resource 3. Only 2 studies (8%) reported randomising the sequence to conduct MOBP and AOBP measurements as well as ABPM; many (at least 12 [46%] studies) had performed MOBP measurement first. Similarly, only 3 studies (12%) reported recruiting patients from primary care populations [25–27]. Most studies were conducted in Western countries, with only three studies conducted in Korea, Iran, and India [28–30]. Nine studies (35%) had fewer than 100 participants. The mean age of participants ranged from 43 to 67.7 years, with their BMI ranging from 26.7 to 30.7 kg/m^2^.

Most studies used BpTRU for AOBP measurement, four studies (15%) used WatchBP, and one study (4%) used Omron HEM-907; similarly, the most commonly used ABPM devices were SpaceLabs devices (SpaceLabs Healthcare; Snoqualmie, USA) (*n* = 12; 46%). AOBP measurements were unattended whenever this was specified (*n* = 21, 81%). Studies were otherwise heterogeneous in the frequency of daytime ABPM, the definition of daytime on ABPM, the number of BP measurements used for ABPM/AOBP/MOBP, MOBP devices, and the person who was responsible for MOBP/AOBP measurements. Most studies did not state the arm used for BP measurement or if the same arm had been used for all three AOBP/MOBP measurements and ABPM; one study used arm with high BP value for AOBP and used both arms for MOBP [29] (Online Resource 3).

### Quality Assessment of the Studies

**Fig. 2:**
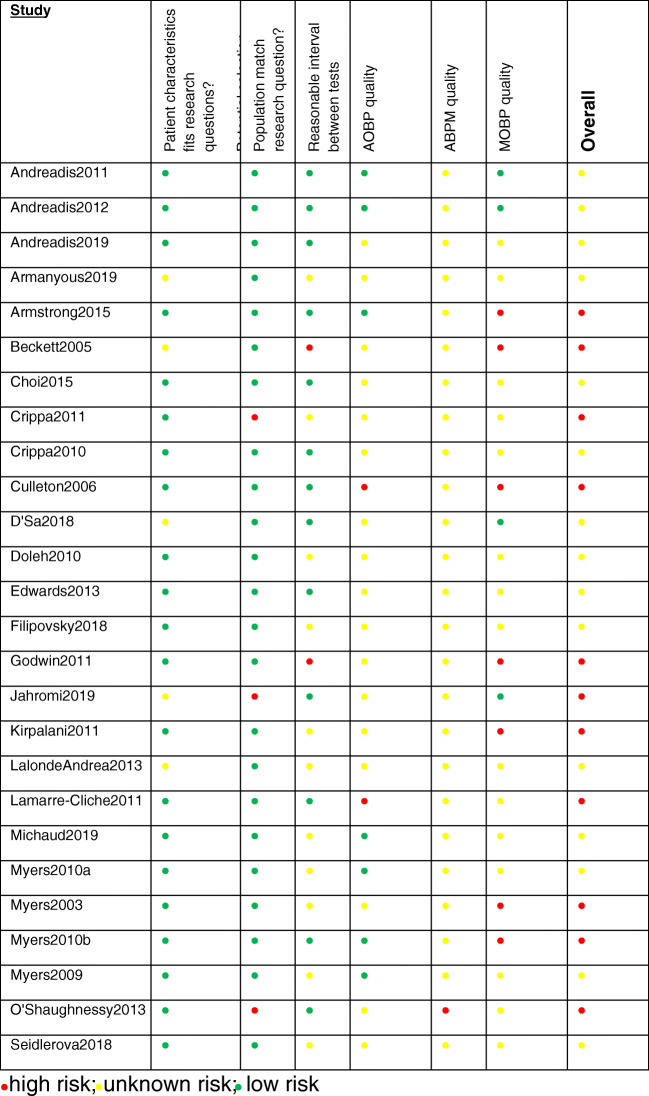
Quality assessment of included studies. high risk; unknown risk; low risk

The quality of AOBP, ABPM, and MOBP in the included studies was individually assessed (Online Resource 4). A summary of quality assessment is presented in Fig. 2.

Almost all studies had not sufficiently described the details of ABPM, which was the reference standard of the current review, and only two studies (7%) had described the criteria for defining valid ABPM results [31, 32]. After extracting the criteria ‘if ABPM was conducted on the non-dominant arm’ (Online Resource 4), it became evident that this was not a valid criterion because researchers might have conducted AOBP and MOBP measurements on the arm with higher BP, which is the recommended BP measurement method for AOBP and MOBP, and would therefore use the same arm for ABPM. Thus, despite this pre-specified criterion, it was not used to evaluate the overall results. However, this did not change the overall results.

Furthermore, 11 studies (37%) had not clearly stated the period between BP measurements [27, 30, 32–40]. Two studies (7%) used MOBP readings retrieved from doctors’ records and BP readings from AOBP/ABPM/MOBP could be weeks to months apart [26, 41].

### Mean Difference in BP between MOBP and AOBP Using ABPM as the Reference Standard

Although this was reported by recent meta-analyses, this is the pre-specified outcome in the PROSPERO. Due to the lack of high-quality study according to preset criteria, subgroup analysis based on study quality was not performed; instead, we performed subgroup analysis for the presence of sequence randomisation of different BP measurements. The detailed results are included in Online Resource 5.

The weighted mean BP of AOBP was not statistically different from that of ABPM for SBP (difference: − 1.32 mmHg; 95% confidence interval [CI]: − 3.56, 0.91; *I*^2^ = 93.4%) and for DBP (difference: − 0.53 mmHg; 95% CI: − 1.59, 0.53; *I*^2^ = 84.4%). The weighted mean BP of MOBP was higher than that of ABPM for SBP (difference: 11.20 mmHg; 95% CI: 7.66, 14.73; *I*^2^ = 97.6%) and for DBP (difference: 4.54 mmHg; 95% CI: 2.81, 6.27; *I*^2^ = 94.7%).

Subgroup analysis of AOBP results indicated that WatchBP had higher weighted mean SBP than ABPM (difference: 5.34 mmHg; 95% CI: 2.1, 8.59; *I*^2^ = 78.2%) and BpTRU had lower weighted mean SBP than ABPM (difference: − 3.11 mmHg; 95% CI: − 5.15, − 1.06; *I*^2^ = 90%). Participants with SBP < 130 mmHg on AOBP measurement had lower weighted mean SBP (difference: − 6.85 mmHg; 95% CI: − 9.4, − 4.3; *I*^2^ = 80.2%) and DBP (difference: − 2.61 mmHg; 95% CI: − 4.24, − 0.99; *I*^2^ = 73.3%) on AOBP measurement than on ABPM. The weighted mean SBP/DBP was similar between studies that used unattended AOBP and studies that did not specify whether the AOBP was attended.

Subgroup analysis of MOBP results revealed that Eastern participants had significantly more white-coat effect than Western participants and that the SBP obtained by MOBP measurement was higher than that obtained by ABPM (difference: 20.26 mmHg; 95% CI: 17.4, 23.11; *I*^2^ = 36.9%). In studies that randomised the BP measurement sequence, MOBP measurement provided mean BP values similar to those obtained by ABPM for both SBP (difference: − 2.45 mmHg; 95% CI: − 8.91, 4.01; *I*^2^ = 80.7%) and DBP (difference: − 3.02 mmHg; 95% CI: − 6.85, 0.8; *I*^2^ = 68.4%). The sensitivity analysis that excluded the study recruiting patients on peritoneal dialysis found similar results (Online Resource 5).

### Sensitivity and Specificity of AOBP/MOBP to Diagnose Elevated BP Using ABPM as the Reference Standard

**Fig. 3:**
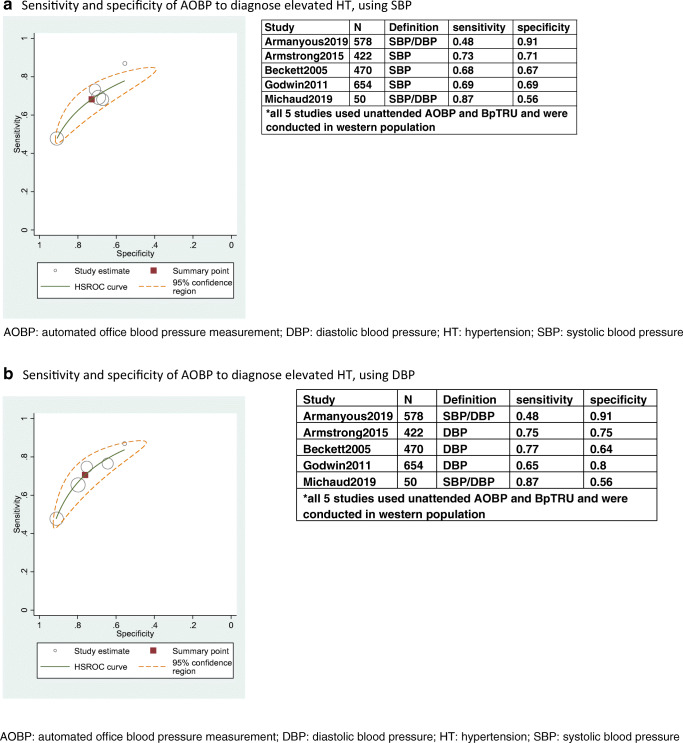
**a** Sensitivity and specificity of AOBP to diagnose elevated HT, using SBP (BP ≥ 135 mmHg), **b** sensitivity and specificity of AOBP to diagnose elevated HT, using DBP (BP ≥ 85 mmHg). AOBP: automated office blood pressure measurement; DBP: diastolic blood pressure; HT: hypertension; SBP: systolic blood pressure

All relevant studies used BpTRU as the AOBP, and the AOBP was unattended. While all studies used 135/85 mmHg as the cut-off for elevated BP on ABPM and AOBP measurement, two studies (Armanyous2019 and Michaud2019) used elevated SBP and/or DBP to define elevated BP [27, 40]. On the contrary, three other studies (Armstrong2015, Beckett2005, and Godwin2011) provided specificity and sensitivity for detecting elevated SBP and elevated DBP separately. We combined the data in two models: the first model used SBP from the three studies and the second one used DBP from the three studies. Specificity and sensitivity from individual studies and the related meta-analysis are shown in Fig. 3.

In the first model, the sensitivity and specificity of AOBP to detect elevated BP were 0.68 (95% CI: 0.58–0.77) and 0.73 (0.59–0.84); in the second model, the sensitivity and specificity of AOBP were 0.71 (95% CI: 0.59–0.80) and 0.76 (95% CI: 0.63–0.86), respectively. Significant heterogeneity was observed in both models (Fig. 3).

As the pooling of specificity and sensitivity required at least 4 studies, the meta-analysis of specificity and sensitivity for MOBP was not possible because there were only 3 studies. The results of these 3 studies are presented in Online Resource 6. The results were heterogeneous: the sensitivity and specificity of MOBP to detect elevated SBP ranged from 6 to 86% and from 24 to 92%, respectively; the sensitivity and specificity of MOBP to detect elevated DBP ranged from 34 to 75% and from 84 to 92%, respectively.

### Proportion of White-Coat and Masked HT by AOBP and MOBP Measurements

**Fig. 4:**
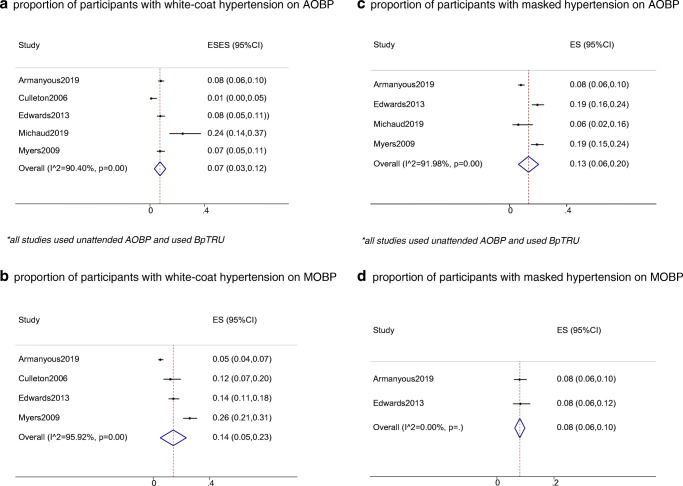
**a** Proportion of participants with white-coat hypertension on AOBP. **b** Proportion of participants with white-coat hypertension on MOBP; **c** proportion of participants with masked hypertension on AOBP; **d** proportion of participants with masked hypertension on MOBP. All studies used unattended AOBP and used BpTRU

All relevant studies used BpTRU as the AOBP, and the AOBP was unattended. The proportion of participants with white-coat HT was 7% (95% CI: 3–12%; *I*^2^ = 90.4%) and 14% (95% CI: 5–23%; *I*^2^ = 95.92%) for AOBP and MOBP, respectively (Fig. 4). The proportion of participants with masked HT was 13% (95% CI: 6–20%; *I*^2^ = 91.98%) and 8% (95% CI: 6–10%; heterogeneity statistics were not reported because there were only 2 studies) for AOBP and MOBP, respectively (Fig. 4).

### Limit of Agreement by Meta-analysing Bland-Altman Statistics

The LOA for SBP and DBP between AOBP and ABPM was − 2.48 mmHg (95% LOA: − 30.52, 25.56) and − 1.42 mmHg (95% LOA: − 17.37, 14.53); the LOA for SBP and DBP between MOBP and ABPM was 10.4 mmHg (95% LOA: − 16.29, 37.09) and 4.19 mmHg (95% LOA: − 14.98, 23.35) (Online Resource 7).

### Publication Bias

**Fig. 5:**
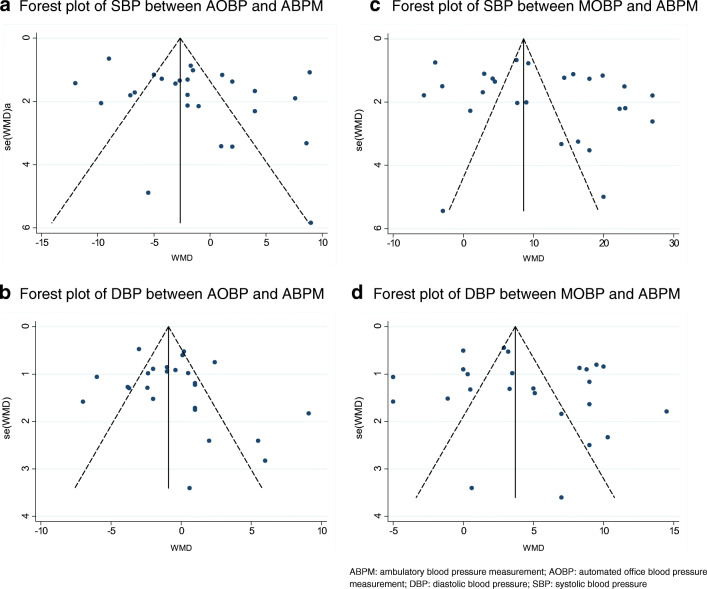
**a** Forest plot of SBP between AOBP and ABPM; **b** forest plot of DBP between AOBP and ABPM; **c** forest plot of SBP between MOBP and ABPM; **d** forest plot of DBP between MOBP and ABPM. ABPM: ambulatory blood pressure measurement; AOBP: automated office blood pressure measurement; DBP: diastolic blood pressure; SBP: systolic blood pressure

Publication bias was assessed using the data regarding mean SBP of AOBP/ABPM/MOBP by visual inspection of the funnel plot (Fig. 5) and Egger’s test, which found no significant small study effect (*p* = 0.133–0.306).

## Discussion

### Main Findings and Comparison with Previous Literature

This study has shown that AOBP could provide closer mean BP estimates to ABPM than the current reference standard of MOBP. The mean SBP and DBP obtained by AOBP measurement were not significantly different from those obtained by ABPM; nonetheless, the mean SBP and DBP obtained by MOBP measurement was on average 11.2 mmHg and 4.5 mmHg higher than ABPM, respectively (Online Resource 5). This occurred when the data were analysed in a group of patients.

In individual patients, the width of LOA was similar between AOBP and MOBP for SBP (56.08 versus 53.38 mmHg, respectively) and was slightly narrower with AOBP than with MOBP for DBP (31.9 versus 38.33 mmHg), although the mean of the LOA was closer to 0 for AOBP than for MOBP, signifying an overall decrease in observed rate of white-coat effect with AOBP. Accordingly, our meta-analysis of the proportion showed that approximately 7% and 14% had white-coat HT on AOBP and MOBP measurements, respectively.

However, it was uncertain from the current data if more individuals will suffer from masked HT on AOBP measurement than on MOBP measurement. With respect to AOBP, the proportion of participants with masked HT was 13% on average (in contrast to 8% for MOBP, although this only included 2 studies) and AOBP underestimated SBP at lower BP range (when AOBP was < 130 mmHg) in the current meta-analysis. Reassuringly, Verberk et al. published a meta-analysis suggesting that the prevalence of masked HT on MOBP measurement was 16.8% (95% CI: 13.0–20.5%) [42]. As the LOA was shifted closer to 0 for AOBP and the width of LOA was similar between AOBP and MOBP, it is possible that there may be an increase in the diagnosis of masked HT when AOBP is used instead of MOBP. This should be further explored in future studies.

The current study also found that AOBP had a sensitivity of about 68–71% and specificity of 73–76% to detect elevated BP. This suggests that although AOBP and ABPM provide the same mean BP values in a group of patients, AOBP cannot replace ABPM and AOBP provides different results in individual patients. The direct comparison between AOBP and MOBP was difficult in this review because results from MOBP were more heterogeneous and did not allow for definite conclusions to be made. This may be because the 3 studies that presented MOBP sensitivity and specificity data were conducted differently—one study retrieved MOBP record from GP record [26], while another study was conducted in 17 patients receiving peritoneal dialysis [22]. A published meta-analysis suggested that the sensitivity and specificity of MOBP to detect elevated BP at the cut-off of 140/90 mmHg was 74.6% (95% CI: 60.7 to 84.8%) and 74.6% (95% CI: 47.9 to 90.4%), respectively, which were similar to our analysis of AOBP [43].

### Comparison with Previous Meta-analyses

The two previously related meta-analyses predominantly compared the mean weighted SBP/DBP obtained by AOBP/MOBP measurements and ABPM (Pappaccogli et al. also included home BP monitoring). These two meta-analyses determined that the mean BP values obtained by AOBP measurement were not statistically different from those obtained by ABPM and that the mean BP values obtained by MOBP measurement were significantly higher than those obtained by ABPM [15, 16]. The current meta-analysis found very similar results. However, to the best of our knowledge, the current study is the first to investigate and compare the specificity and sensitivity, LOA, and proportion of white-coat and masked HT on AOBP and MOBP measurements.

Previous meta-analyses included any studies between AOBP and either ABPM or MOBP [15, 16]. The current study required the same patient to undergo AOBP, MOBP, and ABPM such that results from AOBP and MOBP could be compared among the same group of participants and thus potentially reduce heterogeneity.

### Implications

While many researchers use MOBP as endpoint measurements, AOBP measurement is likely the more appropriate method, as was performed in the SPRINT trial [44], because AOBP measurement can provide a mean BP reading for a group of participants that is more similar to that obtained by ABPM. Although AOBP may reduce the rate of the observed white-coat HT and is potentially cost-saving, direct cost-effective analysis research is required.

In individual patients, the two office measurement methods appear to have otherwise comparable sensitivity and specificity. It is uncertain if AOBP may increase the diagnosis of masked HT, and AOBP underestimate SBP at lower BP range (when AOBP was < 130 mmHg) in the current meta-analysis. As most of the studies included used unattended AOBP (and no studies included in the current meta-analysis reported using attended AOBP), this might prevent wide implementation in general practice due to limitation of space and clinic arrangements. Recent reviews were heterogeneous in their conclusions but attended AOBP may provide higher BP values than unattended AOBP [21, 45, 46].

### Strength and Limitation of This Review and Current Evidence

Out of the three available systematic reviews, the current review was the only one that was pre-registered, and also the only one that assessed quality considering the way AOBP/MOBP/ABPM were performed. The inclusion criteria of the current review were strictest and required the same participants to undergo all AOBP, MOBP, and ABPM. The Chinese database was deliberately searched, although no additional studies were found. In addition, this is the only review that compares AOBP and MOBP further than the weighted mean BP.

However, the investigators could only read English and Chinese; only studies published in English and Chinese were included. However, since most studies published in other language would have an abstract in English, our search should have included them.

However, a few limitations should be discussed. Similar to the other 2 reviews, the results were heterogeneous due to significant clinical heterogeneity—studies used different models of ABPM/MOBP/AOBP and were conducted in different populations. Different studies were diverse in the person to measure BP, the number of BP readings obtained, the arm chosen for BP measurement, the interval between BP measurements, and settings where BP was obtained. In particular, MOBP was obtained diversely by different studies (including obtaining from previous records and manual BP by different healthcare professionals) and many studies did not provide details of MOBP measurements. This high statistical heterogeneity was investigated post hoc by meta-regression with different pre-defined subgroups (Online Resources 5) and demographic data (mean age of participants, percentage of participants with hypertension/on anti-hypertensive medications/mean BMI) (data not shown; all associations were not statistically significant except that the mean difference between MOBP/ABPM SBP was associated with percentage of patients receiving anti-hypertensive medications [beta-coefficient: − 0.177, *p* = 0.03]). If there were more than one significant association found (*p* < 0.05), a meta-regression including all predictors was conducted. However, high residual heterogeneity was not resolved or explained by the meta-regression models (Online Resources 5 and 8). The high heterogeneity weakened our ability to draw definite conclusion and resulted in imprecise effect size estimates.

The current available evidence represented mostly an unfair comparison between AOBP and MOBP because the sequences of BP measurements were not randomised (only randomised in 2 included studies) and at least 46% of the included studies measured MOBP first—it was known that even with the same BP measurement method, BP can decrease by more than 10 mmHg on repeated measurements [47]. In our analysis, there were no significant BP differences between MOBP and ABPM when the BP measurement sequences were randomised (Online Resource 5); although the presence of randomisation did not affect results between AOBP and ABPM—possibly because AOBP was done after MOBP and/or ABPM. Therefore, it is currently unclear if inferior diagnostic performance of MOBP is due to lack of adequate BP sequence randomisation, which can produce differential white-coat effect to MOBP results. Furthermore, it was known that several measurements of MOBP were needed to diagnose elevated BP [48]—but at least three studies only used a single MOBP measurement. These factors would have overestimated the difference between MOBP and ABPM and weakened our conclusion.

Most primary studies used BpTRU. However, BpTRU was no longer produced, and our results suggest that BpTRU and non-BpTRU AOBP may give different BP readings; and BpTRU and other AOBP machines use different measurement algorithm. Similarly, there was a lack of studies in different populations, such as in the Chinese population. Indeed, the behaviour of BP may be different among different ethnicities [49, 50]. Thus, more high-quality studies using WatchBP and HEM-907 with randomised BP measurement sequence and in different ethnicities are needed. Furthermore, there was a lack of studies that directly compared between the three available AOBP with or without comparing to ABPM [51, 52]. Such studies could clarify whether different AOBP gives different results.

### Perspectives

Comparison of mean BP in a group of participants demonstrated that AOBP provided similar BP readings to ABPM—the reference BP measurement method—and MOBP overestimates BP. Available evidence also suggested that AOBP could reduce the rate of the observed white-coat HT, and may be beneficial in clinical practices. The current recommendation, however, is limited by the absence of high-quality studies and the high heterogeneity of our results. More high-quality studies using different AOBP machines and in different population are therefore needed.

## Supplementary Information

ESM 1 Search strategies in different databases (DOCX 13 kb)

ESM 2 Quality assessment criteria (DOCX 16 kb)

ESM 3 List and characteristics of the included studies (DOCX 64 kb)

ESM 4 Quality assessment of AOBP, ABPM, and MOBP according to preset criteria (DOCX 24 kb)

ESM 5 Weighted mean SBP/DBP difference between AOBP/MOBP and ABPM (DOCX 368 kb)

ESM 6 MOBP sensitivity and specificity to detect elevated SBP/DBP (DOCX 27 kb)

ESM 7 Meta-analysis of Bland-Altman statistics (DOCX 20 kb)

ESM 8 Meta-regression (DOCX 16 kb)

## References

[1] Sarafidis PA, Li S, Chen SC, Collins AJ, Brown WW, Klag MJ (2008). Hypertension awareness, treatment, and control in chronic kidney disease. Am J Med.

[2] Moran AE, Odden MC, Thanataveerat A, Tzong KY, Rasmussen PW, Guzman D (2015). Cost-effectiveness of hypertension therapy according to 2014 guidelines. N Engl J Med.

[3] Druss BG, Marcus SC, Olfson M, Tanielian T, Elinson L, Pincus HA (2001). Comparing the national economic burden of five chronic conditions. Health Aff (Millwood).

[4] National Clinical Guideline Centre. Hypertension: clinical management of primary hypertension in adults (NICE clinical guideline 127). London: NICE; 2011. p. 38.

[5] Dolan E, Stanton A, Thijs L, Hinedi K, Atkins N, McClory S (2005). Superiority of ambulatory over clinic blood pressure measurement in predicting mortality: the Dublin outcome study. Hypertension..

[6] Niiranen TJ, Hänninen MR, Johansson J, Reunanen A, Jula AM (2010). Home-measured blood pressure is a stronger predictor of cardiovascular risk than office blood pressure: the Finn-Home study. Hypertension..

[7] Whelton PK, Carey RM, Aronow WS, Casey DE, Collins KJ, Dennison Himmelfarb C (2018). 2017 ACC/AHA/AAPA/ABC/ACPM/AGS/APhA/ASH/ASPC/NMA/PCNA guideline for the prevention, detection, evaluation, and management of high blood pressure in adults: executive summary: a report of the American College of Cardiology/American Heart Association Task Force on Clinical Practice Guidelines. J Am Coll Cardiol.

[8] Xie X, Atkins E, Lv J, Bennett A, Neal B, Ninomiya T (2016). Effects of intensive blood pressure lowering on cardiovascular and renal outcomes: updated systematic review and meta-analysis. Lancet..

[9] Gorostidi M, Vinyoles E, Banegas JR, De La Sierra A (2015). Prevalence of white-coat and masked hypertension in national and international registries. Hypertens Res.

[10] Myers MG, Godwin M, Dawes M, Kiss A, Tobe SW, Kaczorowski J (2010). Measurement of blood pressure in the office: recognizing the problem and proposing the solution. Hypertension..

[11] Myers MG, Godwin M (2012). Automated office blood pressure. Can J Cardiol.

[12] Leung AA, Daskalopoulou SS, Dasgupta K, McBrien K, Butalia S, Zarnke KB (2017). Hypertension Canada’s 2017 guidelines for diagnosis, risk assessment, prevention, and treatment of hypertension in adults. Can J Cardiol.

[13] Myers MG (2014). Eliminating the human factor in office blood pressure measurement. J Clin Hypertens (Greenwich).

[14] Kang YY, Li Y, Huang QF, Song J, Shan XL, Dou Y (2015). Accuracy of home versus ambulatory blood pressure monitoring in the diagnosis of white-coat and masked hypertension. J Hypertens.

[15] Roerecke M, Kaczorowski J, Myers MG (2019). Comparing automated office blood pressure readings with other methods of blood pressure measurement for identifying patients with possible hypertension: a systematic review and meta-analysis. JAMA Intern Med.

[16] Pappaccogli M, Di Monaco S, Perlo E, Burrello J, D’Ascenzo F, Veglio F (2019). Comparison of automated office blood pressure with office and out-off-office measurement techniques. Hypertension..

[17] Williams B, Mancia G, Spiering W, Agabiti Rosei E, Azizi M, Burnier M (2018). 2018 ESC/ESH guidelines for the management of arterial hypertension: the Task Force for the management of arterial hypertension of the European Society of Cardiology and the European Society of Hypertension: the Task Force for the management of arterial hypertension of the European Society of Cardiology and the European Society of Hypertension. J Hypertens.

[18] Andreadis EA, Agaliotis GD, Angelopoulos ET, Tsakanikas AP, Kolyvas GN, Mousoulis GP (2012). Automated office blood pressure is associated with urine albumin excretion in hypertensive subjects. Am J Hypertens.

[19] Andreadis EA, Agaliotis GD, Angelopoulos ET, Tsakanikas AP, Chaveles IA, Mousoulis GP (2011). Automated office blood pressure and 24-h ambulatory measurements are equally associated with left ventricular mass index. Am J Hypertens.

[20] Whiting PF, Rutjes AW, Westwood ME, Mallett S, Deeks JJ, Reitsma JB (2011). QUADAS-2: a revised tool for the quality assessment of diagnostic accuracy studies. Ann Intern Med.

[21] Andreadis EA, Thomopoulos C, Geladari CV, Papademetriou V (2019). Attended versus unattended automated office blood pressure: a systematic review and meta-analysis. High Blood Press Cardiovasc Prev.

[22] O’Shaughnessy MM, Durcan M, Kinsella SM, Griffin MD, Reddan DN, Lappin DW (2013). Blood pressure measurement in peritoneal dialysis: which method is best?. Perit Dial Int.

[23] Roush GC, Fagard RH, Salles GF, Pierdomenico SD, Reboldi G, Verdecchia P (2014). Prognostic impact from clinic, daytime, and night-time systolic blood pressure in nine cohorts of 13 844 patients with hypertension. J Hypertens.

[24] Williams B, Mancia G, Spiering W, Agabiti Rosei E, Azizi M, Burnier M (2018). 2018 ESC/ESH Guidelines for the management of arterial hypertension: the Task Force for the management of arterial hypertension of the European Society of Cardiology and the European Society of Hypertension. J Hypertens.

[25] Crippa G, Cassi A, Bosi M, Fares M (2010). Usefulness of multiple blood pressure measurements using an automated oscillometric monitor (BpTRU) during a campaign for cardiovascular prevention. J Clin Hypertens.

[26] Godwin M, Birtwhistle R, Delva D, Lam M, Casson I, MacDonald S (2011). Manual and automated office measurements in relation to awake ambulatory blood pressure monitoring. Fam Pract.

[27] Michaud A, Lamarre-Cliche M, Cloutier L (2019). Screening for hypertension: an elevated office blood pressure measurement is valuable, adding an automated one is even better. Blood Press Monit.

[28] Choi TY, Rhee M, Kim JH, Namgung J, Lee SY, Cho DK (2015). Multiple office blood pressure measurement with an automated device is superior to blood pressure measured by the doctor in the diagnosis of hypertension: a prospective multicenter study. J Am Coll Cardiol.

[29] Jahromi SE, Haghighi G, Roozbeh J, Ebrahimi V (2019). Comparisons between different blood pressure measurement techniques in patients with chronic kidney disease. Kidney Res Clin Pract.

[30] Kirpalani D, Shah H, Bhabhe A, Kirpalani A (2011). BpTRU-a useful alternative to 24 hour ambulatory blood pressure monitoring in evaluation and management of hypertension in India: PO-285. J Clin Hypertens.

[31] Culleton BF, McKay DW, Campbell NR (2006). Performance of the automated BpTRU measurement device in the assessment of white-coat hypertension and white-coat effect. Blood Press Monit.

[32] Seidlerová J, Gelžinský J, Mateřánková M, Ceral J, König P, Filipovský J (2018). In the aftermath of SPRINT: further comparison of unattended automated office blood pressure measurement and 24-hour blood pressure monitoring. Blood Press.

[33] Armanyous S, Ohashi Y, Lioudis M, Schold JD, Thomas G, Poggio ED (2019). Diagnostic performance of blood pressure measurement modalities in living kidney donor candidates. Clin J Am Soc Nephrol.

[34] Crippa G, Cassi A, Bosi M, Fares M (2011). Usefulness of automated office blood pressure measurement by BPTRU for the diagnosis of resistant hypertension. J Clin Hypertens.

[35] Filipovský J, Seidlerová J, Ceral J, Vysočanová P, Špác J, Souček M (2018). A multicentre study on unattended automated office blood pressure measurement in treated hypertensive patients. Blood Press.

[36] Lalonde AES, Trudeau L, Holcroft C, Tagalakis V, Schiffrin EL (2013). The BpTRU automated blood pressure device: a surrogate for ambulatory blood pressure monitoring?. J Clin Hypertens.

[37] Myers MG (2010). A proposed algorithm for diagnosing hypertension using automated office blood pressure measurement. J Hypertens.

[38] Myers MG, Valdivieso MA (2003). Use of an automated blood pressure recording device, the BpTRU, to reduce the “white coat effect” in routine practice. Am J Hypertens.

[39] Myers MG, Valdivieso M, Kiss A (2009). Use of automated office blood pressure measurement to reduce the white coat response. J Hypertens.

[40] Andreadis EA, Geladari CV, Angelopoulos ET, Kolyvas GN, Papademetriou V (2019). Morning surge and peak morning ambulatory blood pressure versus automated office blood pressure in predicting cardiovascular disease. High Blood Press Cardiovasc Prev.

[41] Beckett L, Godwin M (2005). The BpTRU automatic blood pressure monitor compared to 24 hour ambulatory blood pressure monitoring in the assessment of blood pressure in patients with hypertension. BMC Cardiovasc Disord.

[42] Verberk WJ, Kessels AG, de Leeuw PW (2008). Prevalence, causes, and consequences of masked hypertension: a meta-analysis. Am J Hypertens.

[43] Hodgkinson J, Mant J, Martin U, Guo B, Hobbs FDR, Deeks JJ (2011). Relative effectiveness of clinic and home blood pressure monitoring compared with ambulatory blood pressure monitoring in diagnosis of hypertension: systematic review. BMJ..

[44] Wright JT, Williamson JD, Whelton PK, Snyder JK, Sink KM, SPRINT Research Group (2015). A randomized trial of intensive versus standard blood-pressure control. N Engl J Med.

[45] Myers MG, Sierra A, Roerecke M, Kaczorowski J (2020). Attended versus unattended automated office blood pressure measurement in the diagnosis and treatment of hypertension. J Hypertens.

[46] Kollias A, Stambolliu E, Kyriakoulis KG, Gravvani A, Stergiou GS (2019). Unattended versus attended automated office blood pressure: Systematic review and meta-analysis of studies using the same methodology for both methods. J Clin Hypertens (Greenwich).

[47] Hartley RM, Velez R, Morris RW, D’Souza MF, Heller RF (1983). Confirming the diagnosis of mild hypertension. Br Med J.

[48] Powers BJ, Olsen MK, Smith VA, Woolson RF, Bosworth HB, Oddone EZ (2011). Measuring blood pressure for decision making and quality reporting: where and how many measures?. Ann Intern Med.

[49] Eastwood SV, Tillin T, Chaturvedi N, Hughes AD (2015). Ethnic differences in associations between blood pressure and stroke in South Asian and European men. Hypertension..

[50] Brown MJ (2006). Hypertension and ethnic group. BMJ..

[51] Myers MG, Valdivieso M, Kiss A, Tobe SW (2009). Comparison of two automated sphygmomanometers for use in the office setting. Blood Press Monit.

[52] Myers MG (2017). Automated office blood pressure-incorporating SPRINT into clinical practice. Am J Hypertens.

