# Supplementary material for: Comparison Between Automated Office Blood Pressure Measurements and Manual Office Blood Pressure Measurements—Implications in Individual Patients: a Systematic Review and Meta-analysis
Source: Curr Hypertens Rep. 2021 Jan 15;23(1):4. doi: 10.1007/s11906-020-01118-1 (PMC7810619; doi:10.1007/s11906-020-01118-1)
Supplement: Supplementary file 1 — Search strategies in different databases (DOCX 13 kb) [file 11906_2020_1118_MOESM1_ESM.docx]

**Appendix 1: search strategies in different databases**

Ovid MEDLINE search strategy:

1. Blood Pressure Monitors/ or Blood Pressure Determination/ or Sphygmomanometers/ or Blood Pressure/

2. (sphygmomanomet* or mercury manometer).mp.

3. ((clinic or conventional or manual or manual office or home or office or office-based or in-office or auscultatory or oscillometric or non-invasive or wrist) adj2 (blood pressure or BP)).mp.

4. (MOBP or OBPM or HBPM).mp.

5. 1 or 2 or 3 or 4

6. Blood Pressure Monitoring, Ambulatory/

7. (ambulatory adj (blood pressure or BP)).mp.

8. (MABP or AABP or ABPM).mp.

9. 6 or 7 or 8

10. (AOBP or (automated office or automated) adj (blood pressure or BP)).mp.

11. (HEM-907 or HEM 907 or WatchBP or Watch BP or BPTRU or BP TRU).mp.

12. 10 or 11

13. 5 and 9 and 12

14. limit 13 to "all adult (19 plus years)"

EMBASE search strategy:

1. blood pressure monitor/ or blood pressure measurement/ or sphygmomanometer/ or blood pressure/

2. (sphygmomanomet* or mercury manometer).mp.

3. ((clinic or conventional or manual or manual office or home or office or office-based or in-office or auscultatory or oscillometric or non-invasive or wrist) adj2 (blood pressure or BP)).mp.

4. (MOBP or OBPM or HBPM).mp.

5. 1 or 2 or 3 or 4

6. blood pressure monitoring/

7. (ambulatory adj (blood pressure or BP)).mp.

8. (MABP or AABP or ABPM).mp.

9. 6 or 7 or 8

10. (AOBP or (automated office or automated) adj (blood pressure or BP)).mp.

11. (HEM-907 or HEM 907 or WatchBP or Watch BP or BPTRU or BP TRU).mp.

12. 10 or 11

13. 5 and 9 and 12

14. limit 13 to (adult <18 to 64 years> or aged <65+ years>)

Scopus search strategy:

1. TITLE-ABS-KEY ( "blood pressure monitor*" OR "blood pressure measur*" OR sphygmomanomet* OR "mercury manometer" )

2. TITLE-ABS-KEY ( ( clinic OR conventional OR manual OR home OR office OR office-based OR in-office OR auscultatory OR oscillometric OR non-invasive OR wrist ) PRE/2 ( "blood pressure" OR BP) )

3. TITLE-ABS-KEY ( MOBP OR OBPM OR HBPM )

4. #1 OR #2 OR #3

5. TITLE-ABS-KEY ( “ambulatory blood pressure” OR “ambulatory BP” )

6. TITLE-ABS-KEY ( MABP OR AABP OR ABPM )

7. #5 OR #6

8. TITLE-ABS-KEY ( AOBP OR ( "automated office" OR automated ) PRE/ ( "blood pressure" OR BP ) )

9. TITLE-ABS-KEY ( {HEM-907} OR {HEM 907} OR watchbp OR {Watch BP} OR BPTRU OR {BP TRU} )

10. #8 OR #9

11. #4 AND #7 AND #10

China Academic Journals Full-text Database search strategy:

AB=血壓 and

AB=(測量+監測+量度+檢測) and

AB=(診室+診間+隨診+候診+水銀+MOBP+OBPM) and

AB=(活動式+24小時+二十四小時+攜帶+動態+MABP+AABP+ABPM) and

AB=(自動+AOBP+'HEM-907'+'HEM 907'+WatchBP+'Watch BP'+BPTRU+'BP TRU')
