# Supplementary material for: Comparison Between Automated Office Blood Pressure Measurements and Manual Office Blood Pressure Measurements—Implications in Individual Patients: a Systematic Review and Meta-analysis
Source: Curr Hypertens Rep. 2021 Jan 15;23(1):4. doi: 10.1007/s11906-020-01118-1 (PMC7810619; doi:10.1007/s11906-020-01118-1)
Supplement: Supplementary file 2 — Quality assessment criteria (DOCX 16 kb) [file 11906_2020_1118_MOESM2_ESM.docx]

**Appendix 2 quality assessment**

Under patient selection:

- Could the patient selection have introduced bias? (Was a consecutive or random sample of patients enrolled? Was a case–control design avoided? Did the study avoid inappropriate exclusions?)
- Are there concerns that the included patients do not match the review question?

Under index test – AOBP, the criteria is developed according to latest Canadian guideline (21)

- Was cuff size selection discussed?
- Was the patient seated in a quiet room?
- Was the BP taken every 1 to 2 minutes?
- Was the first measurement validated by a healthcare professional before he/she left the room?

Under index test – OBP, the criteria is developed according to latest ESH guideline (22)

- If equipment other than mercury sphygmomanometer was used, was the machine validated by any international protocol?
- Were at least 2 blood pressure readings taken?
- Was cuff size selection discussed?
- If the BP was manually taken, was the method to take BP discussed and standardized? Were there concerns about the way the BP was taken?
- If the BP was manually taken, were the results from ABPM/AOBP blinded from the observer?

Under reference test – ABPM, the criteria is developed according to latest ESH guideline (22,23):

- Was the ABPM validated and/or recommended by an international society (either stated in the paper or listed on the websites (European guideline or BIHS)?
- Was the ABPM performed on a routine work day?
- Was the frequency of measurement between 15-30 minutes?
- Was ABPM attached to the non-dominant arm?
- Was the appropriate cuff size discussed?
- Was the ABPM result valid? A valid result was either (i) at least 70% of readings were valid during the measurement period OR (ii) At least 20 valid daytime readings
- The guidelines suggested no editing to the ABPM data. If data was edited, was there any concern?
