# Supplementary material for: Comparison Between Automated Office Blood Pressure Measurements and Manual Office Blood Pressure Measurements—Implications in Individual Patients: a Systematic Review and Meta-analysis
Source: Curr Hypertens Rep. 2021 Jan 15;23(1):4. doi: 10.1007/s11906-020-01118-1 (PMC7810619; doi:10.1007/s11906-020-01118-1)
Supplement: Supplementary file 3 — List and characteristics of the included studies (DOCX 64 kb) [file 11906_2020_1118_MOESM3_ESM.docx]

Appendix 3. list and characteristics of included studies

**Studies characteristics.**

| **Study** | **Country** | **Setting** | **Sequence of BP** | **Participants** | **Number of participants** | %  male | %  HT | Mean age | Mean BMI |
| --- | --- | --- | --- | --- | --- | --- | --- | --- | --- |
| Andreadis2011^1^ | Greece | Specialist centre | OBP; ABPM; AOBP | Referred for suspected hypertension by family physicians. Excluded: secondary hypertension, arrhythmia, heart failure, stroke, coronary artery diseases, renal insufficiency, mental diseases, and severe non-cardiovascular disease; patients working night shifts | 90 | 54 | 100 | 54 | 30 |
| Andreadis2012^2^ | Greece | Specialist centre | OBP; ABPM; AOBP | Referred for suspected hypertension by family physicians. Excluded: secondary hypertension, renal insufficiency, DM, systematic infections, recent strenuous physical exercise, substantial psychiatric illness and severe non-cardiovascular disease; patients working night shifts | 162 | 48 | 100 | 53 | 29.9 |
| Andreadis2019^3^ | Greece | Specialist centre | office BP; AOBP; ABPM | patients with high office BP, not on any HT medications; excluded: secondary HT, gross proteinuria, stage IV/V CKD, HR (EF <30%), cancer, serious medical illnesses | 236 | 49 | 100 | 53.5 | 29.9 |
| Armanyous2019^4^ | USA | Specialist centre | NA | living kidney donors | 578 | 44 | 16 | 43 | 27 |
| Armstrong2015^5^ | Canada | Specialist centre | OBP; ABPM; AOBP | Consecutive patients referred for ABPM | 422 | 47.87 | NA | 58.6 | NA |
| Beckett2005^6^ | Canada | Specialist centre | OBP; AOBP; ABPM | All patients with a diagnosis of hypertension who were being treated with antihypertensive medications; excluded: pregnancy and secondary HT | 470 | NA | 100 | NA | NA |
| Choi2015^7^ | Korea | Specialist centre | NA | Diagnosed as hypertension by doctor's manual BP measurement | 266 | NA | NA | NA | NA |
| Crippa2011^8^ | Italy | NA | OBP; AOBP; ABPM | Patients with resistant hypertension | 65 | NA | NA | NA | NA |
| Crippa2010^9^ | Italy | Primary care | OBP; AOBP; ABPM | Patients with unknown, untreated, or uncontrolled hypertension | 68 | 45.59 | 100 | 61 | NA |
| Culleton2006^10^ | Canada | Specialist centre | ABPM; trained nurse OBP; AOBP; GP OBP | Referred by family physicians for ABPM, excluded patients with good BP control on OBP (<140/90mmHg). | 107 | 46.7 | 100 | 57 | NA |
| D'Sa2018^11^ | UK | Specialist centre | AOBP/OBP sequence randomized; both AOBP/OBP then ABPM the both AOBP/OBP | From hypertension clinics and from vascular research clinic | 106 | 50.93 | 52 | 53 | 26.7 |
| Doleh2010^12^ | USA | Specialist centre | NA | NA | 170 | NA | NA | NA | NA |
| Edwards2013^13^ | Canada | Specialist centre | OBP; AOBP; ABPM | Adults with hypertension | 329 | 48.9 | NA | 61.8 | NA |
| Filipovsky2018^14^ | Czech republic | Specialist centre | not fixed | Adults with stable hypertension. Excluded patients with unstable health state, e.g., heart failure or malignancy | 172 | 51.2 | NA | 63.7 | NA |
| Godwin2011^15^ | Canada | Primary care | OBP (from record); AOBP; ABPM | Adults with hypertension and on medications; excluded patients with pregnancy or secondary causes of HT | 654 | 43.4 | 99.2 | 63 | 30.7 |
| Jahromi 2019^16^ | Iran | Specialist centre | NA | Adults with chronic kidney disease stage 3-4; excluded: atrial fibrillation, titration of anti-HT medications in last 1 month | 64 | 60.9 | 39.1 | 59.3 | 28.1 |
| Kirpalani2011^17^ | India | NA | NA | NA | 100 | NA | NA | NA | NA |
| LalondeAndrea2013^18^ | Canada | Specialist centre | NA | NA | 199 | NA | NA | NA | NA |
| Lamarre-Cliche2011^19^ | Canada | Specialist centre | Randomized | Adults with hypertension; excluded: recent changes of medications in 2 weeks or planned medication changes during study period, non-compliance to medications, DM | 99 | 53.47 | 100 | 58.2 | 28.4 |
| Michaud 2019^20^ | Canada | Primary care | office BP; AOBP; ABPM | adults with elevated office BP; excluded: pregnancy; emergency medical conditions; BP >180/110mmHg | 50 | 54 | 20 | 51.9 | 29.5 |
| Myers2010a^21^ | Canada | Specialist centre | NA | NA | 254 | 47.64 | NA | 56.8 | NA |
| Myers2003^22^ | Canada | Specialist centre | OBP by different persons; AOBP; ABPM | Referred to specialist centre for diagnosis or management of hypertension; suspicion of white-coat effect; excluded patients who are unsuitable for ABPM, e.g., atrial fibrillation | 22 | 68.18 | 63.64 | 61 | NA |
| Myers2010b^23^ | Canada | Specialist centre | NA | NA | 300 | 45.33 | NA | 60 | NA |
| Myers2009^24^ | Canada | Specialist centre | OBP; AOBP; ABPM | Referred by their family physician for 24-h ABPM | 309 | 44.98 | NA | NA | NA |
| O'Shaughnessy2013^25^ | Ireland | Specialist centre | NA | All patients on peritoneal dialysis; excluded patients who aged less than 18 yo, PD treatment less than 3 months, changes in medications/PD in preceding 2 weeks | 17 | 71 | 100 | 54.2 | NA |
| Seidlerova2018^26^ | Czech republic | NA | NA | Adults with essential hypertension on stable dose of medications | 98 | 52 | 100 | 67.7 | NA |

ABPM: ambulatory blood pressure monitoring; AOBP: automated office blood pressure measurements; BP: blood pressure; DM: diabetes mellitus; GP: general practitioner; HT: hypertension; NA: not applicable (because not reported); MOBP: manual office blood pressure measurements; PD: Peritoneal Dialysis; UK: United Kingdom; USA: United States of America

**AOBP characteristics in included studies**

| **Study** | **AOBP model** | **Person responsible** | **Attended/unattended** | **Waiting time before measuring BP** | **Arm used** | **No of readings** | **Intervals of measurement** |
| --- | --- | --- | --- | --- | --- | --- | --- |
| Andreadis2011 | microlife WatchBP Office | research doctor | Unattended | yes. 5 minutes | both arms | 6 | 1 minutes |
| Andreadis2012 | microlife WatchBP Office | NA | Unattended | yes. 5 minutes | both arms | 6 | 1 minutes |
| Andreadis2019 | microlife WatchBP Office | NA | Unattended | Yes. 5 minutes | Both arms | 6 | 1 minutes |
| Armanyous2019 | BpTRU | clinic staff | unattended | NA | NA | 5 | 1 minutes |
| Armstrong2015 | BpTRU | research nurse | Unattended | NA | arm with higher BP | 5 | 2 minutes |
| Beckett2005 | BpTRU | research nurse | Unattended | yes. 5 minutes | non-dominant | 5 | either 1 or 2 minutes |
| Choi2015 | WatchBP | Nurse | NA | NA | both arms | ‘3 times every 3 days visits’ | NA |
| Crippa2011 | BpTRU | NA | Unattended | NA | NA | 5 | NA |
| Crippa2010 | BpTRU | NA | NA | NA | NA | 5 | 1 minutes |
| Culleton2006 | BpTRU | research nurse | Unattended | yes. 5 minutes | NA | 5 | 5 minutes |
| D'Sa2018 | OmronHEM-907 | study operator | Unattended | yes. 5 minutes | non-dominant arm | 3 | 2 minutes |
| Doleh2010 | BpTRU | NA | NA | NA | NA | 5 | 1 minutes |
| Edwards2013 | BpTRU | research nurse | Unattended | yes. 5 minutes | arm with higher BP | 5 | 1 minutes |
| Filipovsky2018 | BpTRU | NA | Unattended | yes. 5 minutes | NA | 5 | 1 minutes |
| Godwin2011 | BpTRU | research nurse | Unattended | yes. 5 minutes | non-dominant arm | 5 | either 1 or 2 minutes |
| Jahromi 2019 | WatchBP | Nurse | unattended | NA | Arm with higher BP | 3 | 1 minutes |
| Kirpalani2011 | BpTRU | NA | NA | NA | NA | 5 | 2 minutes |
| LalondeAndrea2013 | BpTRU | NA | NA | NA | NA | 5 | NA |
| Lamarre-Cliche2011 | BpTRU | nurse specialist | Unattended | yes. 5 minutes | NA | 5 | 1 minutes |
| Michaud 2019 | BpTRU | Nurse | unattended | NA | Left arm | 6 | 1 minutes |
| Myers2010a | BpTRU | technician | Unattended | no standardized period of rest | NA | 5 | 1 or 2 minutes interval |
| Myers2003 | BpTRU | NA | Unattended | yes. 3 minutes | NA | 2 times each visits; for 2 visits | 2 minutes |
| Myers2010b | BpTRU | research staff | Unattended | NA | NA | 5 | 1 minutes |
| Myers2009 | BpTRU | NA | Unattended | NA | NA | 5 | 1 or 2 minutes interval |
| O'Shaughnessy2013 | BpTRU | PD nurse | Unattended | NA | NA | 5 | 2 minutes |
| Seidlerova2018 | BpTRU | NA | Unattended | No | NA | 5 | 1 minute |

AOBP: automated office blood pressure; BP: blood pressure; NA: not applicable (not reported); PD: peritoneal dialysis

**ABPM characteristics in included studies**

| **Study** | **ABPM model** | **Daytime measurement frequency** | **Daytime/night-time definition** | **Valid result definition** | **Editing of results** | **Arm used** |
| --- | --- | --- | --- | --- | --- | --- |
| Andreadis2011 | Microlife WatchBPO3 | 15 minutes | sleep diary | NA | readings of SBP <70mmHg or >260mmHg and/or DBP <40mmHg or >150mmHg were disregarded. | NA |
| Andreadis2012 | Microlife WatchBPO3 | 15 minutes | sleep diary | NA | NA | NA |
| Andreadis2019 | Microlife WatchBPO3 | 20 minutes | sleep diary | NA | NA | NA |
| Armanyous2019 | NA | 20 minutes | NA | NA | NA | NA |
| Armstrong2015 | SpaceLab model 90207 | 30 minutes | Fixed period | NA | NA | arm with higher BP |
| Beckett2005 | A&D TM 2430 | 15 minutes | Fixed period | NA | NA | NA |
| Choi2015 | Mobil-O-Graph | NA | NA | NA | NA | NA |
| Crippa2011 | NA | NA | NA | NA | NA | NA |
| Crippa2010 | NA | NA | NA | NA | NA | NA |
| Culleton2006 | SpaceLab model 90207 | NA | sleep diary | less than 15% error | as per manufacture | NA |
| D'Sa2018 | Mobil-O-Graph | 30 minutes | only wear at least 8 hours during daytime | NA | NA | non-dominant arm |
| Doleh2010 | NA | NA | NA | NA | NA | NA |
| Edwards2013 | SpaceLab 90207 | 15 minutes | NA | NA | NA | arm with higher BP |
| Filipovsky2018 | SpaceLab devices | NA | NA | NA | NA | NA |
| Godwin2011 | A&D TM 2430 | NA | NA | NA | NA | NA |
| Jahromi 2019 | NA | 30 minutes | sleep diary | NA | NA | NA |
| Kirpalani2011 | NA | NA | NA | NA | NA | NA |
| LalondeAndrea2013 | NA | NA | NA | NA | NA | NA |
| Lamarre-Cliche2011 | SpaceLabs 90207 | 30 minutes | NA | NA | NA | NA |
| Michaud 2019 | SpaceLabs OnTrak 9027 | 30minutes | NA | NA | NA | NA |
| Myers2010a | SpaceLab 90207 | 15 minutes | patient diary | NA | NA | NA |
| Myers2003 | SpaceLabs 90207 | 15 minutes | Fixed period | NA | NA | NA |
| Myers2010b | SpaceLabs 90207 | 15 minutes | patient diary | NA | NA | NA |
| Myers2009 | SpaceLabs 90207 | 15 minutes | patient diary | NA | NA | NA |
| O'Shaughnessy2013 | SpaceLabs 90207 | 20 minutes | Fixed period | 14 valid daytime measurements and 7 night-time measurements | NA | NA |
| Seidlerova2018 | SpaceLabs devices | 15 minutes | Fixed period | at least 20 daytime and 10 night-time valid BP readings | NA | NA |

ABPM: ambulatory blood pressure monitoring; DBP: diastolic blood pressure; NA: not applicable (not reported); SBP: systolic blood pressure

**MOBP characteristics in included studies**

| **Study** | **MOBP measurement methods** | **No of readings** | **Arm used** | **Person who measured BP** |
| --- | --- | --- | --- | --- |
| Andreadis2011 | Microlife WatchBP Office | 3 | NA | research doctor |
| Andreadis2012 | Microlife BP A100 | 3 | NA | research doctor |
| Andreadis2019 | Microlife WatchBP Office | 6 | both arms | NA |
| Armanyous2019 | automated oscillometric aneroid sphygmoanometer | NA | NA | trained clinic staff |
| Armstrong2015 | BpTRU | 1 | arm with higher BP | research nurse |
| Beckett2005 | Unstandardized from GP record | 3 | NA | record from GP |
| Choi2015 | NA | NA | NA | doctors |
| Crippa2011 | NA | NA | NA | NA |
| Crippa2010 | NA | 2 | NA | NA |
| Culleton2006 | Mercury sphygmomanometer by a research nurse, not discussed what method used by GP | 2 for nurse, unknown for doctor | NA | research nurse, GP |
| D'Sa2018 | OmronHEM-907 | 2 | non-dominant arm | study operator |
| Doleh2010 | NA | NA | NA | primary care physicians |
| Edwards2013 | NA | 3 | arm with higher BP | research nurse |
| Filipovsky2018 | NisseiDM-3000 and N desk mode | 3 | NA | physician in the clinic |
| Godwin2011 | From GP practice record | 3 | NA | physician in the clinic |
| Jahromi 2019 | mercury sphygmomanometer | NA | both arms | NA |
| Kirpalani2011 | NA | 1 | NA | NA |
| LalondeAndrea2013 | Mercury aneroid sphygmomanometer | 2 | NA | nurse clinician |
| Lamarre-Cliche2011 | Mercury sphygmomanometer | 3 | NA | research nurse |
| Michaud 2019 | BpTRU | NA | left arm | nurse clician |
| Myers2010a | From GP practice record | NA | NA | patient family physician |
| Myers2003 | Last routine reading taken by patients' family physician; research technician 2x mercury sphygmomanometer readings | NA | NA | NA |
| Myers2010b | Mercury sphygmomanometer | 2 | NA | research staff |
| Myers2009 | From referral GP; 2 readings by technicians using mercury sphygmomanometer | 2 for technician; unknown for referral doctor | NA | GP/technician |
| O'Shaughnessy2013 | Vital Signs monitor 300 | Unstandardized | NA | PD nurse |
| Seidlerova2018 | NisseiDM-3000 | 3 | NA | physician |

BP: blood pressure; GP: general practitioner; NA: not applicable (not reported); PD: peritoneal dialysis

References:

1. Andreadis EA, Agaliotis GD, Angelopoulos ET, Tsakanikas AP, Chaveles IA, Mousoulis GP. Automated office blood pressure and 24-h ambulatory measurements are equally associated with left ventricular mass index. Am J Hypertens. 2011;24(6):661–666.

2. Andreadis EA, Agaliotis GD, Angelopoulos ET, Tsakanikas AP, Kolyvas GN, Mousoulis GP. Automated office blood pressure is associated with urine albumin excretion in hypertensive subjects. Am J Hypertens. 2012; 25(9)25:969–973. doi: 10.1038/ajh.2012.76

3. Andreadis EA, Geladari C V., Angelopoulos ET, Kolyvas GN, Papademetriou V. Morning Surge and Peak Morning Ambulatory Blood Pressure Versus Automated Office Blood Pressure in Predicting Cardiovascular Disease. High Blood Press Cardiovasc Prev. 2019; 26(3):209–215. doi: 10.1007/s40292–019–00315–7.

4. Armanyous S, Ohashi Y, Lioudis M, Schold JD, Thomas G, Poggio ED, Augustine JJ. Diagnostic Performance of Blood Pressure Measurement Modalities in Living Kidney Donor Candidates. Clin J Am Soc Nephrol. 2019; 14(5):738-746. doi:10.2215/CJN.02780218

5. Armstrong D, Matangi M, Brouillard D, Myers MG. Automated office blood pressure-being alone and not location is what matters most. Blood Press Monit. 2015; 20(4):204-8. doi: 10.1097/MBP.0000000000000133.

6. Beckett L, Godwin M. The BpTRU automatic blood pressure monitor compared to 24 hour ambulatory blood pressure monitoring in the assessment of blood pressure in patients with hypertension. BMC Cardiovasc Disord. 2005; 5(1):18.5. doi: 10.1186/1471-2261-5-18.

7. Choi T-Y, Rhee M, Kim J-H, Namgung J, Lee SY, Cho D-K, Kim S-Y, Kim J-Y, Park J-S, Park S-M. MULTIPLE OFFICE BLOOD PRESSURE MEASUREMENT WITH AN AUTOMATED DEVICE IS SUPERIOR TO BLOOD PRESSURE MEASURED BY THE DOCTOR IN THE DIAGNOSIS OF HYPERTENSION: A PROSPECTIVE MULTICENTER STUDY. J Am Coll Cardiol. 2015; 65 (10). doi: 10.1016/S0735-1097(15)61429-2.

8. Crippa G, Cassi A, Bosi M, Fares M. USEFULNESS OF AUTOMATED OFFICE BLOOD PRESSURE MEASUREMENT BY BPTRU FOR THE DIAGNOSIS OF RESISTANT HYPERTENSION. J Clin Hypertens. 2011;13:A124.

9. Crippa G, Cassi A, Bosi M, Fares M. Usefulness of multiple blood pressure measurements using an automated oscillometric monitor (BpTRU) during a campaign for cardiovascular prevention. J Clin Hypertens. 2010;12:A18–147.

10. Culleton BF, McKay DW, Campbell NR. Performance of the automated BpTRU^TM^ measurement device in the assessment of white-coat hypertension and white-coat effect. Blood Press Monit. 2006; 11(1)11:37–42. doi: 10.1097/01.mbp.0000189794.36230.a7.

11. D’Sa L, Senaratne N, Woodcock-Smith J, Miles KM, Wilkinson IB, McEniery CM. Evaluation of the Omron HEM-907 automated blood pressure device: comparison with office and ambulatory blood pressure readings. Hypertens Res. 2019;42(1):52-58. doi: 10.1038/s41440-018-0120-7. 12. Doleh T, Pohl M, Butler R, Schrelber M, Rafey M. Comparison of automated sphygmomanometer (BPTRU) measurements performed in a 5 minute cycle with mean awake ambulatory blood pressure. J Clin Hypertens. 2010;12(Supp1):A73–74.

13. Edwards C, Hiremath S, Gupta A, McCormick BB, Ruzicka M. BpTRUth: Do automated blood pressure monitors outperform mercury? J Am Soc Hypertens. 2013; 7(6):448-53. doi: 10.1016/j.jash.2013.07.002.

14. Filipovský J, Seidlerová J, Ceral J, Vysočanová P, Špác J, Souček M, Řiháček I, Mateřánková M, König P, Rosolová H. A multicentre study on unattended automated office blood pressure measurement in treated hypertensive patients. Blood Press. 2018; 27(4)27:188–193. doi: 10.1080/08037051.2018.1425606.

15. Godwin M, Birtwhistle R, Delva D, Lam M, Casson I, MacDonald S, Seguin R. Manual and automated office measurements in relation to awake ambulatory blood pressure monitoring. Fam Pract. 2011; 28(1):110-7. doi: 10.1093/fampra/cmq067.

16. Jahromi SE, Haghighi G, Roozbeh J, Ebrahimi V. Comparisons between different blood pressure measurement techniques in patients with chronic kidney disease. Kidney Res Clin Pract. 2019; 38(2)38:212–219. doi: 10.23876/j.krcp.18.0109.

17. D.A. K, H.K. S, A.S. B. BPTRU - A useful alternative to 24 hour ambulatory blood pressure monitoring in evaluation and management of hypertension in India. J Clin Hypertens. 2011; 137;Suppl 1:S17–S18.

18. Lalonde Andrea ES, Luc T, Christina H, Vicky T, Schiffrin Ernesto L. The BPTRU automated blood pressure device: A surrogate for ambulatory blood pressure monitoring? J Clin Hypertens. 2013; 15: Suppl 1.

19. Lamarre-Cliché M, Cheong NNG, Larochelle P. Comparative Assessment of Four Blood Pressure Measurement Methods in Hypertensives. Can J Cardiol. 2011; 27(4):455-60. doi: 10.1016/j.cjca.2011.05.001

20. Michaud A, Lamarre-Cliche M, Cloutier L. Screening for hypertension: an elevated office blood pressure measurement is valuable, adding an automated one is even better. Blood Press Monit. 2019;24(3):123–129. doi: 10.1097/MBP.0000000000000382.

21. Myers MG. A proposed algorithm for diagnosing hypertension using automated office blood pressure measurement. J Hypertens. 2010; 28(4)28:703–708. doi: 10.1097/HJH.0b013e328335d091

22. Myers MG, Valdivieso MA. Use of an automated blood pressure recording device, the BpTRU, to reduce the “white coat effect” in routine practice. Am J Hypertens. 2003; 16(6)16:494–497. doi: 10.1016/s0895–7061(03)00058-x

23. Myers M, Valdivieso M, Chessman M, Kiss A. Can sphygmomanometers designed for self-measurement of blood pressure in the home be used in office practice?. Blood Press Monit. 2010; 15(6):300-4. doi: 10.1097/MBP.0b013e328340d128.

24. Myers MG, Valdivieso M, Kiss A. Use of automated office blood pressure measurement to reduce the white coat response. J Hypertens. 2009; 27(2)27:280–286. doi: 10.1097/HJH.0b013e32831b9e6b.

25. O’Shaughnessy MM, Durcan M, Kinsella SM, Griffin MD, Reddan DN, Lappin DW. Blood pressure measurement in peritoneal dialysis: which method is best? Perit Dial Int. 2013; 33(5)33:544–551. doi: 10.3747/pdi.2012.00027.

26. Seidlerová J, Gelžinský J, Mateřánková M, Ceral J, König P, Filipovský J. In the aftermath of SPRINT: further comparison of unattended automated office blood pressure measurement and 24-hour blood pressure monitoring. Blood Press. 2018; 27(5)27:256–261. doi: 10.1080/08037051.2018.1454258.
