# Supplementary material for: Comparison Between Automated Office Blood Pressure Measurements and Manual Office Blood Pressure Measurements—Implications in Individual Patients: a Systematic Review and Meta-analysis
Source: Curr Hypertens Rep. 2021 Jan 15;23(1):4. doi: 10.1007/s11906-020-01118-1 (PMC7810619; doi:10.1007/s11906-020-01118-1)
Supplement: Supplementary file 4 — Quality assessment of AOBP, ABPM, and MOBP according to preset criteria (DOCX 24 kb) [file 11906_2020_1118_MOESM4_ESM.docx]

**Appendix 4: quality assessment of AOBP, ABPM and MOBP according to pre-set criteria**

**AOBP**

| **Study** | **Quiet room for measurement?** | **1-2 minutes measurement intervals?** | **1^st^ reading validated?** | **Cuff size discussed?** |
| --- | --- | --- | --- | --- |
| Andreadis2011 | Low | low | low | Low |
| Andreadis2012 | Low | low | low | Low |
| Andreadis2019 | low | low | low | unclear |
| Armanyous2019 | unclear | low | unclear | Unclear |
| Armstrong2015 | Low | low | low | Low |
| Beckett2005 | Low | low | low | Unclear |
| Choi2015 | Unclear | unclear | Unclear | Unclear |
| Crippa2011 | Low | unclear | Low | Unclear |
| Crippa2010 | Unclear | low | Unclear | Unclear |
| Culleton2006 | Low | high | Unclear | Low |
| D'Sa2018 | Low | low | Unclear | Low |
| Doleh2010 | Unclear | low | Low | Unclear |
| Edwards2013 | Low | low | Low | Unclear |
| Filipovsky2018 | Low | low | Unclear | Unclear |
| Godwin2011 | Low | low | Low | Unclear |
| Jahromi2019 | unclear | low | unclear | unclear |
| Kirpalani2011 | Unclear | low | Unclear | Unclear |
| LalondeAndrea2013 | Unclear | unclear | Unclear | Unclear |
| Lamarre-Cliche2011 | unclear | low | high | Unclear |
| Michaud2019 | low | low | low | low |
| Myers2010a | Low | low | Low | Low |
| Myers2003 | Low | low | unclear | Unclear |
| Myers2010b | Low | low | low | Low |
| Myers2009 | Low | low | low | Low |
| O'Shaughnessy2013 | Low | low | low | Unclear |
| Seidlerova2018 | Low | low | unclear | Unclear |

**ABPM**

| **Study** | **Validated ABPM?** | **Done on workday?** | **Frequency of ABPM 15-30minutes?** | **ABPM on non-dominant arm?** | **ABPM results valid according to guideline?** | **Concern about editing of ABPM results** | **Cuff size discussed for ABPM** |
| --- | --- | --- | --- | --- | --- | --- | --- |
| Andreadis2011 | Low | Low | low | Unclear | unclear | low | low |
| Andreadis2012 | Low | Unclear | low | Unclear | unclear | low | low |
| Andreadis2019 | low | low | low | unclear | unclear | unclear | unclear |
| Armanyous2019 | unclear | Unclear | low | unclear | unclear | unclear | Unclear |
| Armstrong2015 | Low | Unclear | low | Unclear | unclear | unclear | unclear |
| Beckett2005 | Low | Low | low | Low | unclear | unclear | Unclear |
| Choi2015 | Low | Unclear | unclear | Unclear | unclear | unclear | unclear |
| Crippa2011 | Unclear | Unclear | unclear | Unclear | unclear | unclear | unclear |
| Crippa2010 | Unclear | Unclear | unclear | Unclear | unclear | unclear | unclear |
| Culleton2006 | Low | Low | unclear | Unclear | low | unclear | low |
| D'Sa2018 | Low | Low | low | Low | unclear | unclear | low |
| Doleh2010 | Unclear | Unclear | unclear | Unclear | unclear | unclear | unclear |
| Edwards2013 | Low | Unclear | low | Unclear | unclear | unclear | unclear |
| Filipovsky2018 | Unclear | Unclear | unclear | Unclear | unclear | unclear | unclear |
| Godwin2011 | Low | Unclear | unclear | Unclear | unclear | unclear | unclear |
| Jahromi2019 | unclear | unclear | low | unclear | unclear | unclear | unclear |
| Kirpalani2011 | Unclear | Unclear | unclear | Unclear | unclear | unclear | unclear |
| LalondeAndrea2013 | Unclear | Unclear | unclear | Unclear | unclear | unclear | unclear |
| Lamarre-Cliche2011 | Low | Unclear | low | Unclear | unclear | unclear | unclear |
| Michaud2019 | unclear | unclear | low | low | unclear | unclear | unclear |
| Myers2010a | Low | Unclear | low | Unclear | unclear | unclear | unclear |
| Myers2003 | Low | Low | low | Unclear | unclear | unclear | unclear |
| Myers2010b | Low | Unclear | low | Unclear | unclear | unclear | low |
| Myers2009 | Low | Unclear | low | unclear | unclear | unclear | unclear |
| O'Shaughnessy2013 | Low | Low | low | Unclear | high | unclear | unclear |
| Seidlerova2018 | Unclear | Unclear | low | Unclear | low | unclear | unclear |

**MOBP**

| **Study** | **Valid BP machine?**  **If mercury used, results blinded (if done after AOBP/ABPM)? Is the process standardized?** | **At least 2 measurements used?** | **Cuff size discussed?** |
| --- | --- | --- | --- |
| Andreadis2011 | Low | low | Low |
| Andreadis2012 | Low | low | Low |
| Andreadis2019 | low | Low | Unclear |
| Armanyous2019 | low | unclear | Unclear |
| Armstrong2015 | Low | High | Unclear |
| Beckett2005 | Unclear | Low | High |
| Choi2015 | Unclear | unclear | Unclear |
| Crippa2011 | Unclear | unclear | Unclear |
| Crippa2010 | Unclear | Low | Unclear |
| Culleton2006 | High | Unclear | Unclear |
| D'Sa2018 | Low | Low | Low |
| Doleh2010 | Unclear | Unclear | Unclear |
| Edwards2013 | Low | Low | Unclear |
| Filipovsky2018 | Unclear | Low | Unclear |
| Godwin2011 | High | Low | Unclear |
| Jahromi2019 | Low | Low | Low |
| Kirpalani2011 | Unclear | High | Unclear |
| LalondeAndrea2013 | Unclear | Low | Unclear |
| Lamarre-Cliche2011 | unclear | Low | Low |
| Michaud2019 | Low | Unclear | low |
| Myers2010a | unclear | unclear | Unclear |
| Myers2003 | Low | High | Unclear |
| Myers2010b | high | Low | Low |
| Myers2009 | Unclear | Unclear | Unclear |
| O'Shaughnessy2013 | low | Unclear | Unclear |
| Seidlerova2018 | Low | Low | Unclear |
