# Supplementary material for: Comparison Between Automated Office Blood Pressure Measurements and Manual Office Blood Pressure Measurements—Implications in Individual Patients: a Systematic Review and Meta-analysis
Source: Curr Hypertens Rep. 2021 Jan 15;23(1):4. doi: 10.1007/s11906-020-01118-1 (PMC7810619; doi:10.1007/s11906-020-01118-1)
Supplement: Supplementary file 5 — Weighted mean SBP/DBP difference between AOBP/MOBP and ABPM (DOCX 368 kb) [file 11906_2020_1118_MOESM5_ESM.docx]

**Weighted mean difference of SBP between AOBP and ABPM (subgroup: AOBP model)**

Meta-regression of BpTRU versus non-BpTRU:
- beta-coefficient: -7.28, p= 0.03
- residual I^2^ = 89.14%

**Weighted mean difference of SBP between AOBP and ABPM (subgroup: ethnicity)**

Meta-regression of subgroups:
- beta-coefficient: -7.71, p= 0.024
- residual I^2^ = 90.75%

**Weighted mean difference of SBP between AOBP and ABPM (subgroup: presence of BP sequence randomization)**

Meta-regression of subgroups:
- beta-coefficient: -3.54, p= 0.4
- residual I^2^ = 93.63%

**Weighted mean difference of SBP between AOBP and ABPM (subgroup: if AOBP SBP ≥130mmHg)**

Meta-regression of subgroups:
- beta-coefficient: 7.26, p= 0.002
- residual I^2^ = 87.73%

**Weighted mean difference of SBP between AOBP and ABPM (subgroup: AOBP unattended/not specified**)

Meta-regression of subgroups:
- beta-coefficient: -1.39, p= 0.603
- residual I^2^ = 93.20%

**Weighted mean difference of SBP between AOBP and ABPM (subgroup: AOBP model)**

(Sensitivity analysis by removing study (n=1) involving patients with peritoneal dialysis)

**Weighted mean difference of DBP between AOBP and ABPM (subgroup: AOBP model)**

Meta-regression of BpTRU versus non-BpTRU:
- beta-coefficient: -0.26, p= 0.882
- residual I^2^ = 84.99%

**Weighted mean difference of DBP between AOBP and ABPM (subgroup: ethnicity)**

Meta-regression of subgroups:
- beta-coefficient: -0.65, p= 0.811
- residual I^2^ = 85.00%

**Weighted mean difference of DBP between AOBP and ABPM (subgroup: presence of BP sequence randomization)**

Meta-regression of subgroups:
- beta-coefficient: -4.34, p= 0.087
- residual I^2^ = 83.95%

**Weighted mean difference of DBP between AOBP and ABPM (subgroup: if AOBP SBP ≥130mmHg)**

Meta-regression of subgroups:
- beta-coefficient: 2.85, p= 0.056
- residual I^2^ = 79.46%

**Weight mean difference of DBP between AOBP and ABPM (subgroup: AOBP unattended or not specified)**

Meta-regression of subgroups:
- beta-coefficient: -2.24, p= 0.186
- residual I^2^ = 80.29%

**Weighted mean difference of DBP between AOBP and ABPM (subgroup: AOBP model)**

(Sensitivity analysis by removing study (n=1) involving patients with peritoneal dialysis)

**Weighted mean difference of SBP between MOBP and ABPM (subgroup: person responsible for BP measurement)**

**Weighted mean difference of SBP between MOBP and ABPM (subgroup: ethnicity)**

Meta-regression of subgroups:
- beta-coefficient: -9.79, p= 0.111
- residual I^2^ = 97.31%

**Weighted mean difference of SBP between MOBP and ABPM (subgroup: presence of BP sequence randomization)**

Meta-regression of subgroups:
- beta-coefficient: -14.68, p= 0.042
- residual I^2^ = 97.53%

**Weighted mean difference of SBP between MOBP and ABPM (subgroup: instrument used for measurement)**

Meta-regression of subgroups:
- beta-coefficient: 3.78, p= 0.368
- residual I^2^ = 97.42%

**Weighted mean difference of SBP between MOBP and ABPM (subgroup: person responsible for BP measurement)**

(Sensitivity analysis by removing study (n=1) involving patients with peritoneal dialysis)

**Weighted mean difference of DBP between MOBP and ABPM (subgroup: person responsible for BP measurement)**

**Weighted mean difference of DBP between MOBP and ABPM (subgroup: ethnicity)**

Meta-regression of subgroups:
- beta-coefficient: -2.17, p= 0.577
- residual I^2^ = 94.9%

**Weighted mean difference of DBP between MOBP and ABPM (subgroup: presence of BP sequence randomization)**

Meta-regression of subgroups:
- beta-coefficient: -8.242, p= 0.023
- residual I^2^ = 94.44%

**Weighted mean difference of DBP between MOBP and ABPM (subgroup: instrument used for measurement)**

Meta-regression of subgroups:
- beta-coefficient: 1.87, p= 0.382
- residual I^2^ = 93.95%

**Weighted mean difference of DBP between MOBP and ABPM (subgroup: person responsible for BP measurement)**

(Sensitivity analysis by removing study (n=1) involving patients with peritoneal dialysis)
