# Supplementary material for: Comparison Between Automated Office Blood Pressure Measurements and Manual Office Blood Pressure Measurements—Implications in Individual Patients: a Systematic Review and Meta-analysis
Source: Curr Hypertens Rep. 2021 Jan 15;23(1):4. doi: 10.1007/s11906-020-01118-1 (PMC7810619; doi:10.1007/s11906-020-01118-1)
Supplement: Supplementary file 6 — MOBP sensitivity and specificity to detect elevated SBP/DBP (DOCX 27 kb) [file 11906_2020_1118_MOESM6_ESM.docx]

Appendix 6a: MOBP sensitivity and specificity to detect elevated SBP (BP≥140mmHg)

Sensitivity

Specificity

Appendix 6b: MOBP sensitivity and specificity to detect elevated DBP (BP≥90mmHg)

Sensitivity

Specificity
