# Supplementary material for: Comparison Between Automated Office Blood Pressure Measurements and Manual Office Blood Pressure Measurements—Implications in Individual Patients: a Systematic Review and Meta-analysis
Source: Curr Hypertens Rep. 2021 Jan 15;23(1):4. doi: 10.1007/s11906-020-01118-1 (PMC7810619; doi:10.1007/s11906-020-01118-1)
Supplement: Supplementary file 7 — Meta-analysis of Bland-Altman statistics (DOCX 20 kb) [file 11906_2020_1118_MOESM7_ESM.docx]

Appendix 7: Meta-analysis of Bland-Altman statistics

AOBP SBP - ABPM SBP

| *Study* | *n* | *Mean difference*  *(mmHg)* | *95% Lower LOA*  *(mmHg)* | *95% higher LOA*  *(mmHg)* |
| --- | --- | --- | --- | --- |
| Andreadis2011 | 90 | 4.1 | -20.2 | 28.4 |
| Andreadis2012 | 162 | 7.9 | -16.8 | 32.6 |
| Andreadis2019 | 236 | 3.1 | -26.2 | 32.4 |
| Culleton2006 | 107 | -9.7 | -35.04 | 15.64 |
| D'Sa2018 | 106 | -2 | -23.56 | 19.56 |
| Edwards2013 | 329 | -3.2 | -31 | 33 |
| Filipovsky2018 | 172 | -4.3 | -25.86 | 17.26 |
| Godwin2011 | 654 | -1.8 | -30.7 | 27.1 |
| Jahromi 2019 | 64 | 8.6 | -25.4 | 42.6 |
| LalondeAndrea2013 | 199 | -12 | -40.77 | 16.77 |
| Lamarre-Cliche2011 | 99 | -6.9 | -32.1 | 18.3 |
| Myers2010a | 254 | -2.8 | -28.1 | 22.6 |
| Myers2009 | 309 | -2 | -31.4 | 27.4 |
| Seidlerova2018 | 98 | -6.7 | -32.3 | 18.9 |
| **Summary** | **2879** | **-2.48** | **-30.52** | **25.56** |
| *Summary (excluding LalondeAndrea2013)* | *2680* | *3.54* | *-26.16* | *29.70* |

**only LalondeAndrea2013 did not specify if the AOBP is unattended, sensitivity analysis by excluding LalondeAndrea2013 is presented*

AOBP DBP – ABPM DBP

| Study | N | *Mean difference*  *(mmHg)* | *95% Lower LOA*  *(mmHg)* | *95% higher LOA*  *(mmHg)* |
| --- | --- | --- | --- | --- |
| Andreadis2011 | 90 | 1.1 | -14.4 | 17 |
| Andreadis2019 | 236 | 0.3 | -15.8 | 16.4 |
| Culleton2006 | 107 | -3.9 | -18.9 | 11.1 |
| D'Sa2018 | 106 | -7 | -26.6 | 12.6 |
| Edwards2013 | 329 | -2.4 | -17.9 | 13.1 |
| Filipovsky2018 | 172 | 0.5 | -13.024 | 14.024 |
| Godwin2011 | 654 | 0.24 | -17.3 | 17.7 |
| Jahromi 2019 | 64 | 5.5 | -12.3 | 23.2 |
| LalondeAndrea2013 | 199 | -6 | -21.84 | 9.84 |
| Lamarre-Cliche2011 | 99 | -1.9 | -15.42 | 11.62 |
| Myers2010a | 254 | -1.1 | -16.3 | 14 |
| Myers2009 | 309 | -2 | -17.68 | 13.68 |
| Seidlerova2018 | 98 | -2.4 | -18.4 | 13.6 |
| **Summary** | **2717** | **-1.42** | **-17.37** | **14.53** |
| *Summary (excluding LalondeAndrea2013)* | *2518* | *2.12* | *-14.90* | *17.02* |

**only LalondeAndrea2013 did not specify if the AOBP is unattended, sensitivity analysis by excluding LalondeAndrea2013 is presented*

MOBP SBP – ABPM SBP

| *Study* | *N* | *Mean difference*  *(mmHg)* | *95% Lower LOA*  *(mmHg)* | *95% higher LOA*  *(mmHg)* |
| --- | --- | --- | --- | --- |
| Andreadis2011 | 90 | 27.63 | -22.87 | 78.13 |
| Andreadis2012 | 162 | 30.5 | 0.3 | 60.7 |
| Crippa2010 | 68 | 18 | -5.52 | 41.52 |
| Culleton2006 | 107 | 7.7 | -19.19 | 34.59 |
| D'Sa2018 | 106 | 0 | -27.44 | 27.44 |
| Edwards2013 | 329 | 4.8 | -27.8 | 37.4 |
| Filipovsky2018 | 172 | 4.2 | -19.516 | 27.9 |
| Godwin2011 | 654 | 7.6 | -23.1 | 38.6 |
| Jahromi 2019 | 64 | 16.4 | -13.7 | 46.6 |
| Lamarre-Cliche2011 | 99 | -5.43 | -29.23 | 18.4 |
| Myers2010a | 254 | 14.4 | -15.6 | 44.2 |
| Myers2009 | 309 | 18 | -17.28 | 53.28 |
| Seidlerova2018 | 98 | 2.8 | -8.5 | 0.5 |
| **Summary** | **2512** | **10.4** | **-16.29** | **37.09** |

MOBP DBP -ABPM DBP

| *Study* | *N* | *Mean difference*  *(mmHg)* | *95% Lower LOA*  *(mmHg)* | *95% higher LOA*  *(mmHg)* |
| --- | --- | --- | --- | --- |
| Andreadis2011 | 90 | 9.4 | -20.9 | 39.8 |
| Crippa2010 | 68 | 10 | -5.68 | 25.68 |
| Culleton2006 | 107 | 5.1 | -16.1 | 26.3 |
| D'Sa2018 | 106 | -5 | -22.64 | 12.64 |
| Edwards2013 | 329 | 0.35 | -17.88 | 18.578 |
| Filipovsky2018 | 172 | 3.5 | -11.788 | 18.788 |
| Godwin2011 | 654 | 2.9 | -15.9 | 21.8 |
| Jahromi 2019 | 64 | 10.3 | -11 | 31.7 |
| Lamarre-Cliche2011 | 99 | 0.98 | -13.82 | 15.78 |
| Myers2010a | 254 | 8.3 | -11.3 | 27.7 |
| Myers2009 | 309 | 10 | -11.56 | 31.56 |
| Seidlerova2018 | 98 | 0.5 | -16.5 | 17.5 |
| **Summary** | **2350** | **4.19** | **-14.98** | **23.35** |
