# Supplementary material for: Comparison Between Automated Office Blood Pressure Measurements and Manual Office Blood Pressure Measurements—Implications in Individual Patients: a Systematic Review and Meta-analysis
Source: Curr Hypertens Rep. 2021 Jan 15;23(1):4. doi: 10.1007/s11906-020-01118-1 (PMC7810619; doi:10.1007/s11906-020-01118-1)
Supplement: Supplementary file 8 — Meta-regression (DOCX 16 kb) [file 11906_2020_1118_MOESM8_ESM.docx]

**Appendix 8: meta-regression to investigate heterogeneity**

**Meta-regression for AOBP SBP**

Residual I^2^: 84.87%

|  | Beta-coefficient | p-value | 95% confidence interval |  |
| --- | --- | --- | --- | --- |
| Ethnicity | -5.31 | 0.093 | -11.574 | 0.953 |
| BpTRU vs non-BpTRU | -5.803 | 0.017 | -10.45 | -1.156 |
| SBP of ABPM | 0.174 | 0.175 | -0.832 | 0.431 |
| constant | -15.72 | 0.359 | -50.536 | 19.105 |

**Meta-regression for MOBP SBP**

Residual I^2^: 94.38%

|  | Beta-coefficient | p-value | 95% confidence interval |  |
| --- | --- | --- | --- | --- |
| Percentage of patients on anti-hypertensive medications | -0.196 | 0.008 | -0.321 | -0.714 |
| BP measurement sequence randomization | -17.1914 | 0.043 | -33.63 | -0.751 |
| constant | 23.69 | <0.001 | 15.531 | 31.850 |
